# Academy of Stomatology

**Published:** 1903-09

**Authors:** 

**Affiliations:** Academy of Stomatology


### ACADEMY OF STOMATOLOGY.

A REGULAR meeting of the Academy of Stomatology of Philadelphia was held at its rooms, 1731 Chestnut Street, on the evening of Tuesday, February 24, 1903, the President, Dr. R. Hamill D. Swing, in the chair.

A paper was read by Dr. Myer L. Rhein, of New York, entitled "The Technique of Approximal Restorations with Gold in Posterior Teeth."

(For Dr. Rhein's paper, see page 652.)

A paper was read by Dr. A. C. Eglin, of Philadelphia, entitled "The Removable Bridge as a Conservator of Teeth."

(For Dr. Eglin's paper, see page 663.)

DISCUSSION ON DR. RHEIN'S PAPER.

*Dr. S. H. Guilford.*—I am sure we have all been very highly pleased and edified by Dr. Rhein's paper. I can do nothing but commend it in its general aspects. He has told us a great deal that is entirely true. While the general outline is similar to what most of us practice, there are some points in which I cannot exactly agree with him.

I have here a plaster tooth model with a cavity, somewhat as he described, filled with modelling clay. As to the preparation of the cavity, Dr. Rhein took a position intermediate between those of Webb and Black. Dr. Webb prepared the cervical margin of the cavity so as to conform to the natural cavity; in other words, the form naturally given by the decay; whereas, Dr. Black makes a flat cervical wall. Dr. Rhein holds to the idea of using the cervical wall to resist strain, as claimed by Dr. Black. Whatever the form of the cervical wall when the filling is carried over and anchored on the occlusal surface, no amount of strain is going to make it revolve or move from its place. A filling of this kind is held in place by the shape of the cavity. In the model I have made the cervical wall a cross between a regular curve and a flat surface, but not entirely flat like Black's. I prepare the side walls in such a way as not to depend upon them for anchorage. By the use of the matrix we avoid the necessity for grooves in the lateral walls.

If we pack gold, keeping it highest in the centre, we have an acute or, at least, a right angle formed by the gold and the wall, whereas, we want obtuse angles. In order to obtain an obtuse angle, I carry the filling along the sides a little in advance of the centre. You may say that this is apt to draw it away from the sides, but I have not found it so. I object to cutting cavities of this type with parallel walls, because it entails the loss of too much good tooth-structure.

Dr. Rhein spoke of the abuse of the matrix. According to his statement I have been a user of the matrix for many years, because in most compound cavities I can apply it for the filling of the cavity and for the temporary support of the filling. In using the matrix we can tighten or loosen it to any extent, and thus contour the filling to a greater or less degree at any point.

To assist in starting the filling Dr. Black and Dr. Johnson make deep undercuts at the linguo-cervico-axial and the buccocervico-axial angles, and pack the gold until the entire cervical wall is covered. Dr. Rhein places a little cement, then adds gold to that. There may be no objection to cement, but personally I prefer to have a gold filling throughout. If phosphate of zinc is needed to protect the sensitive part of the tooth, I should use it, but as anchorage I do not need it.

Dr. Rhein spoke of the wall of the enamel along the cervical margin. It is said that there decay recurs most frequently. By his plan,—starting the gold at one side with a little cement and carrying it across,—I do not think he is as certain of protecting that wall as he thinks he is. The edge should be covered absolutely and the gold malleted down. This is easily done by inserting a pellet of gold with tweezers between the matrix and the cervical margin and turning it over the margin. You can place as many pieces of gold as necessary in this way, packing them down as you go. When we get the gold down in perfect contact with the margin the flexible matrix will yield, and in that way all margins are made more than full. There would be no more objection to filling one portion at a time than there would be to placing the matrix and removing it. The gold can be packed in a reasonable time, and when the cavity is filled you simply take a small knife or bistoury and cut off the gold until you have the margins entirely clear. After this you smooth them with a Rhein's trimmer and finish with the polishing strip.

I have used the electric mallet for over thirty years. I had one of the first made by Dr. Bonwill, and many others since. I do not think I have introduced a filling in years in which I have not used it part of the time, except possibly when demonstrating the use of non-cohesive gold.

*Dr. Ralph B. Reitz*, of New York.—In most things operative Dr. Rhein and I are in accord. I have always used the Bonwill mechanical mallet, however, and believe that practically the same results can be obtained with it and with less danger of getting out of repair. If the work be well done, it matters little what mallet is used. I am always willing to take off my hat and make my best bow to such work as Dr. Rhein, Dr. Gardiner, and Dr. Keffer do with the electric mallet. These are mentioned because fillings in my own mouth done by these men with the electric mallet have stood the test. While I rely mainly on the No. 3 Bonwill mechanical mallet, I find occasional use for the Bosworth universal mallet, which is a right-angle mallet. By its use good condensation can be produced over distal walls without the free cutting necessary for any straight mallet. The cutting required in some of these cases to secure free access for the straight mallet is little short of mutilation, and to my mind is uncalled for. I have never cut a pit for starting a filling since I have been in practice. When the shape of the cavity does not lend itself readily to the secure anchoring of the first pieces of gold, I use cement as described by Dr. Rhein, not waiting for it to become thoroughly hard, but go right ahead with my malleting. The cervical margin gives me very little trouble, unless it is near to or under the gum, when I follow the plan outlined by Dr. Guilford. The edges are burnished with springy instruments which I keep expressly for this purpose. It has always been my custom to take whatever time is necessary, regardless of the much shorter time which might be required by another to do the same operation. I do not believe I could be content in filling teeth otherwise.

*Dr. H. A. Clemment.*—I do not think it is necessary for a man to tie himself down to any particular mallet. I seldom use the matrix, but when I do I think I get better margins. The man who uses the electric mallet can do the work much more easily, and the gold is much better condensed. Some of the most beautiful work is done by the use of the automatic mallet. I have been using the electric mallet for eighteen years in conjunction with the automatic mallet, hand-pressure, or anything else which meets the indication. When a patient does not like the electric mallet I use something else, but I use the electric mallet when I can.

*Dr. F. D. Gardiner.*—I wish to thank Dr. Rhein very much for what he has given us. The paper is exactly along the lines upon which I hoped he would write. I agree with him in all the essential particulars. I shape my cavities perhaps a little differently, but I work along the same general methods, because I believe the principle is correct. I believe that a flat cervical wall will detain much better than a rounded one. We should not lose sight of the fact that if a filling has a proper mechanical seat, the lateral walls are relieved of much of the strain that would otherwise be imposed upon them. I think these lateral walls should always be bevelled outward instead of at right angles. Like Dr. Guilford, I would prefer to have the walls of the cavities bevelled so as to give greater protection to the walls of the tooth without weakening. If you bevel the cavity walls outward, it is not necessary to enlarge the cavity in the dentine to so great an extent. It is easier on these lateral walls to insert the filling if these walls are perfectly straight. Otherwise I think we leave a weak occlusal margin likely to be broken away.

The result is the one thing to be borne in mind. Ideal results can be obtained after the method described by Dr. Rhein.

*Dr. E. C. Rice.*—There is one point I would like to bring out: I have been a careful student of Dr. Black's method, and I think it is a mistake to say that he speaks of grooves in the base of his prepared cavity. As I have understood the matter, he uses a right angle on both sides at the base of the cavity, and depends entirely upon the spreading principle of his gold. He cuts his gold in strips and places one end in one corner of the cavity and then grasps the gold again at a point at a greater distance than the width of the cavity, places that in the opposite corner, and condenses the central portion.

*Dr. Charles R. Turner.*—I have most heartily to commend the finishing of the cervical margin at the time that Dr. Rhein recommends that it be done. This is a most vital portion of the filling, and, in spite of the excellent trimmers which he has given us, it is quite a difficult matter to get a nice finish of the gold up to a bevelled margin at this point. I also think that the use of cohesive gold throughout the filling is much to be desired. I believe that there is no question that we may be easily deceived by the quality of the non-cohesive gold placed at this portion of the cavity. If we have a substantial foundation, such as we can get with cohesive gold, we have more reasonable expectation of long life for the filling.

*Dr. James Truman.*—It seems to me that we dwell too much upon men rather than upon principles. We are talking all the time of Varney, Webb, Black, Johnson, and Wedelstaedt. These men simply carried out the principles of filling teeth as they understood them. When I saw Dr. Guilford fill over the cervical wall of his model cavity, I was reminded that Dr. Townsend taught us the same thing in a little different way. There is practically nothing new in it. I cannot understand how any man can put oxyphosphate of zinc in the cavity, place a piece of gold on top of it, and call it a good mechanical piece of work. It strikes me that there is some risk in the method, as we do not know what effect the phosphate of zinc has upon the pulp. I believe the proper mechanical method of starting a filling is that used prior to Webb, of making pits. The filling is then worked in from the very start.

The methods adopted recently with regard to extension are to my mind only partially correct. It is possible that extension may prevent, but it may do exactly the reverse of that. There are no weaker points than the cervical border where moisture is constantly present.

*Dr. William Trueman.*—I have never been convinced that we can make gold so thoroughly solid by any form of filling as if melted in. For that reason I object to stopping in the midst of a filling. I think it always leaves a weak union. I look upon Webb as a most thorough operator and as a most thorough condenser of gold. He filled two front teeth of mine. After a time one broke, and the gold was found to be one-eighth of an inch thick. One gave way in three years, and another in ten. I judge not only from the work, but from the fact that it took him a week to fill four teeth, and on two days he worked from half-past eight o'clock in the morning until five in the afternoon. The gold was one homogeneous mass and as solid as if passed through a rolling-mill. That, I think, is not properly considered in estimating the tensile strength of a gold filling. When it is packed in with an electric mallet it is under strain. I am pleased to note in Dr. Rhein's description of the method of filling that he continues the bevel from the bottom to the top of the cavities, and that he does not depend entirely upon anchorage in occlusal surfaces, because, as more stress is brought to bear upon that point, it will stretch out. I have seen a number of contour fillings break off, probably because the gold was packed towards the buccal wall. There are points in the paper that all should value, while, at the same time, we may not adopt all of them.

*Dr. Rhein* (closing).—I thank you for the kind consideration you have accorded such a worn subject as this. I regret that no one has seen fit to take up the defence of a combination of cohesive and non-cohesive filling for such cavities as these, a method that, so far as I can judge from the writings in dentistry, seems to be the most prominent one in use through the West and Northwest for rapidly restoring the contour surfaces of posterior teeth.

I have a short sentence in my paper in which I say the method I speak of is a delineation of one method of doing this work, and naturally speak of my own method. I recognize, as every professional man must, that all work of this kind is done by all kinds of methods, and I should not attempt to criticise the methods of men eminently successful in their work, but I do claim that the principles I have enunciated make it possible to insert fillings in the quickest, easiest manner, with less strain upon the patient and operator, while producing more enduring results than are possible by any other method. I commend it to young men who have not become wedded to any form of practice.

From what has been said regarding the shape of the cavity, it seems to me that I have not been thoroughly understood, and that Black's ideas have not been thoroughly comprehended. Dr. Guilford, in speaking of cavity preparations, certainly does not recognize the points I attempted to make clear. I agree most heartily with Dr. Gardiner when he says that the preparation of the cavity, as shown by Dr. Guilford, is, in his opinion, absolutely insecure for the preservation of the walls. I tried very hard to make pertinent that while I believed in right-angle preparation of the walls from the floor of the cavity, as laid down by Black, there is an outer bevel of the margin extending around the entire outer periphery of the cavity. While the bevel is much smaller in width in some places than in others, I claim that the continuous strength of the bulk depends upon perfect protection of the enamel-rods. That was always the teaching of Webb. Black and Johnson paid little attention to this point. I recognize the great advance we have made in laying stress upon this principle of the flat nature of the dentinal floor at the cervix.

I never leave the margins parallel. The more extensive the restoration the more I diverge from parallelism, but bevel as I reach the occlusal portion in order that the gold going over that surface shall be of sufficient thickness to withstand an enormous amount of mastication. The main objection to the shaping of Dr. Guilford's cavity is that the enamel-rods are not sufficiently protected. My own belief is that one of the chief causes of the principles of extreme extension has been due to this lack of bevelling the margins. Drs. Guilford and Truman have spoken in criticism of the method of starting the filling. After the use of cement for eighteen years I claim that it is beyond criticism. The amount of cement is about that held on the point of a pin. One of the most valuable points claimed for my work is the thin film of cement around the surface of the dentinal wall. The cement should never extend upon the margins of the cavity, and should be of so small an amount that it would possess no detrimental action and yet be sufficient to cement a minute piece of gold. Dr. Guilford may have the skill to finish his margins after filling, but after filling to the occlusal portion I can never finish the concavity with the same ease and dexterity as when the cavity is only one-half or three-quarters filled, or as I can do it when there is no tooth adjacent.

The clinical history given by Dr. William Trueman of the splitting of his tooth to my mind only proves the truth of the teachings of Webb in this respect and the remarks which I made to-night. We all know that the exceptions prove the rule in everything.

When in finishing the filling of a tooth at a subsequent sitting I have found that the gold would not unite, it was proof to me that there was some imperfection in the previous manipulations, and it is much better for the patient that I made the discovery after having filled the third of the tooth than if the work were completed and then a flaw discovered. I challenge Dr. Trueman or any gentleman to separate at the point of cohesion a filling put in in the manner I have described. The whole principle rests upon the perfect welding together of the gold as taught by Webb.

Dr. Reitz has brought with him a strip of gold to prove what I believe to be a fact,—that its tensile strength is greater than the melted ingot. With the hand-pressure we can weld the gold together as perfectly as with the electric mallet. I do not believe that Dr. Black and Dr. Johnson are responsible for some of the statements we heard regarding their methods of shaping and filling cavities.

With all due respect to Professor Truman, and with all regard that I have for him, I take exception to the statement that we have not progressed in work of this nature in the last twenty-five years. Before the use of cohesive foil and the time he speaks of they knew how to save teeth with gold fillings. Such things Webb speaks of, but we have no evidence of any ability to produce such contour restoration as seen to-day.

*Dr. James Truman.*—I would just like to say a word in regard to what Dr. Rhein has stated. One would naturally suppose that Webb introduced cohesive gold. This, however, was used long before Webb came on the platform. When I was a teacher of operative dentistry Dr. Webb attended my lectures, although graduated from another school. I taught at that time cohesive gold from the beginning to the ending. That was long before Webb's reputation was made. What I meant to say is that the principle of filling teeth was well understood, not only prior to Webb, but, as well, up to the present time. I do not see that there has been any great advance in the operation of filling teeth for the last twenty-five years.

DISCUSSION ON DR. EGLIN'S PAPER.

*Dr. E. C. Kirk.*—This matter of crown- and bridge-work has become so definitely specialized, and in ways quite apart from the lines of my own work, that I hardly feel competent to speak on the subject. I have, however, been very much impressed with the exhibition in one particular,—the element of conservatism. I think every dentist except the man who has gone wild on the subject of bridge-work must have recoiled from the almost wholesale sacrifice of sound teeth. From the little knowledge I have on the subject I concur thoroughly in the belief that the natural crown as a support for bridge-work is better than anything else, because with it we escape the irritation of peridental membrane by ill-fitting bands. From a mechanical stand-point it is evident that there is also less destruction of the tooth-structure. It seems characteristic of all new methods that they are crude, and that after long experience and application we eliminate these crudities and reach something like a rational working-plan. My impression is that the method advocated by the essayist is a step towards legitimate use of that which is a good thing in its place.

*Dr. A. P. Fellows.*—I agree with the essayist that we ought to have a great deal more conservatism in the matter of bridge-work, and one of the ways of being conservative is to save crowns. In this method, while there are strong points, there may also be some weak ones. In the lower bridge shown the placing of a spur upon one of the adjoining teeth would, I think, cause wear of the enamel, and in course of time would promote decay and necessitate crowning. The essayist speaks of the bridge being in use for two or three years. I do not think a patient would care to go to the trouble of having a bridge put in to last only two or three years. I believe that if the molar and cuspid had been crowned in the ordinary way, and fixed firmly, that it would last three times as long, without any trouble at all. I believe that under ordinary conditions a tooth well crowned will last longer than that same tooth if it were not crowned. For instance, in one piece shown there is a bicuspid root which seems to be a very serviceable root, indeed, but it will not support three crowns well even though it has a support on each of the adjoining teeth. It is a question to my mind whether that is really a proper way of handling that kind of a case. I believe that we ought to get all possible utility out of work without losing the crown or endangering the piece of work, but if necessary to the solidity of the work, I believe it is a wise thing to sacrifice that crown without hesitation. Still due consideration should be given before a crown is cut away. I believe the greater number of crown-workers are very careful to get as good results as they can.

*Dr. H. A. Clemment.*—I see no advantage in removable bridge-work, but rather a disadvantage. I have never seen a satisfactory removable bridge. I cannot understand how it is possible to keep a pin in the root without some moisture, which means decay, so that you must crown later. I think a patient is apt not to take good care of a removable bridge, and must come back to the dentist to have it put back again. If the tooth is filled so that when the pin is put in there is a slight space left at the distal side, so that it can be thoroughly cleaned, there will be no need of a removable bridge.

In the lower jaw back of the second bicuspid I only use the occlusal surfaces. When it becomes necessary to sacrifice a crown to give a patient comfort for a number of years, I never hesitate to do it. I act upon this principle from having had such work done in my own mouth. I have known of cases which have been satisfactory for a number of years and free from accumulations of food. This I think is more satisfactory than carrying a removable bridge around in the pocket.

*Dr. Eglin* (closing).—I do not think Dr. Fellows quite understood my remarks regarding the spur. This rests in the gold filling, and if the gold filling is properly put in there is no reason for further decay at that point. The doctor assumes that the bridge which has been in for two years is now ready to come out, but it is as good to-day as when put in, and looks as if it might be good for several years to come.

In answer to Dr. Clemment about putting in the tube, it is cemented in, and after this the needle around which the tube has been made is inserted and a gold filling put in. Any portion of the enamel with which the split pin might come into contact would then be covered with gold, and there is therefore no question of a leak.

The idea of carrying a removable bridge in the pocket should not condemn the method. It may condemn the work, but not the method.

Otto E. Inglis,

*Editor Academy of Stomatology.*

